# First Dentition

**Published:** 1871-05

**Authors:** H. L. Sage

**Affiliations:** Bridgeport, Conn.


DENTAL REGISTER.

Vol. XXV.] MAY, 1871. [No. 5.

### Communications.

### FIRST DENTITION.

BY H. L. SAGE, BRIDGEPORT, CONN.

Read before the Connecticut State Dental Association.

The care of the mouth during the eruption of the temporary teeth, and the management of the diseases common to this period, have been so generally considered as out of the province of the dental surgeon, that they have received but little notice, either as an interesting branch of study, or as requiring his practical attention; and so far as the so-called diseases of dentition are concerned, considering his present status, it is proper that their treatment should fall where it legitimately belongs—to the medical practitioner.

But, as an object of study, the growth and development of the deciduous teeth, and the phenomena connected therewith, should be as familiar to the dentist as it is possible to make them, as well as the means of alleviating the morbid conditions sometimes attendant upon the process of "cutting" them—and this study is rendered doubly interesting to every member of the profession who has his own little ones, as he watches the changes in the oral cavity during this critical period—for critical it is, to a large proportion of children—probably not from the mere fact that all the functions concerned in the evolution of the teeth are so actively engaged, but from the direct relation which subsists between the rapid development of *every* part of the system at this period of life, and the production of disease.

As above stated, the care of the developing teeth and surrounding structures during first dentition, has not as yet, been confided to the hands of the dentist, from the fact that he has not yet made it, or desired to make it, a part of his specialty, by qualifying himself therefor, by a proper degree of attention to the local and sympathetic disturbances accompanying a morbid condition of the nervous and vascular system—the consequence of the above cited causes in subjects constitutionally feeble. Even after the first set of teeth is fully developed, he is only occasionally consulted, and then perhaps, under the apprehension, often groundless, that their removal is necessary. In rare cases, he is called upon to fill them, but usually not until caries has progressed so far as to expose the pulp, or involve the surrounding tissues.

The assertion that the treatment of the diseases of dentition do not fall within the field of practice of the dentist, although true in the main, may perhaps, admit of some qualification by considering them in their proper division of local and sympathetic, for if the former, he should be able to occasionally afford relief, and to mitigate wholly or in part, the severity of the morbid phenomena not unfrequently attendant upon the processes concerned in dentition.

Let us enquire into the meaning of the terms *local* and *sympathetic* as they relate to the subject under consideration. Prof. Bond, defines them thus: "*local* diseases of dentition, are those which affect the teeth, gums and mouth; *sympathetic*, those which manifest themselves in other organs." These are influenced by various causes, among which may be mentioned, the degree of local irritation caused directly by the advancement of the teeth, the amount of constitutional vigor possessed, and which determines in a great measure, the susceptibility of the child thereto, not to speak of the age as having *its* effect, and also the degree of rapidity with which nature performs her functions of growth, development and absorption ; for the capillaries being naturally stimulated to increased activity, may, by the over development of a part, pass the boundaries of the normal process of growth, thus inducing diseased action. But being natural processes, they do not necessarily lead to abnormal conditions, and will not, if the child is perfectly healthy ; for such is the rapidity with which changes occur in infancy, that many of the symptoms accompanying dentition *are* natural, and not the result of disease. Any organ in a state of activity, induces in a corresponding measure a determination of blood to the seat of action, and thus it is, with the gums and subjacent tissues, at this stage of life.

In a healthy subject, an increased flow of saliva is usually attendant upon the process in certain stages, or a moderately lax state of the bowels, thus allaying or preventing the serious consequences which might otherwise ensue. Therefore, it is only to remove the latter when they are excited in a greater or less degree, that any interference is required, at the hands of the medical or dental practitioner.

The "name is legion," of the diseases which have been cited as dependent upon dentition as a predisposing cause, but neither time, space or patience, will admit of a specification of "all the ills" that infancy "is heir to," as being a direct or indirect consequence of an attempt to perfect an organization having in itself the seeds of disease, ready to germinate when the conditions favorable thereto present themselves. "The influence of normal dentition in producing disease and causing death," says Jocobi, "is certainly not such as is often supposed. even by well educated medical men."

One of the most serious complications of dentition, is a gastro-intestinal disorder which never appears at any other period of life—though what connection it has with the process, it is not easy to determine, but that it comes under the head of *sympathetic*, is manifest.

This disease, cholera-infantum, is an abnormal condition of the mucous follicles of the intestines, and perhaps, it may be indirectly incited by irritation of the mucous membrane of the mouth, caused by teething, which extends in its influence to the parts adjacent or subjacent thereto, and continuous therewith—the stomach and bowels sharing in the exalted sensibility.

All the parts concerned in nutrition, at this epoch, are in a high state of activity, and the mucous follicles of the intestines, whose function it is to secrete the fluids concerned in digestion, yield them at this time in great abundance—which, thus far, is a natural and healthful process. Should this secretion become excessive, however, diarrhœa is the consequence, and if allowed to go too far, it becomes complicated with other derangements of the system, and if the exciting causes are not removed, they unite in producing that so often fatal disease of the child's second summer, cholera-infantum. The first year of the infants life, being characterized by the greatest activity in the process of development, the degree becoming more moderate as the child advances to the fourth or fifth year, it is then most subject to violent attacks of disease, and hence we observe the greatest mortality in children during that period. The brain perhaps, undergoes the most rapid changes of any one organ of the body, and it is not surprising that it becomes the seat of disease, when any predisposing cause manifests itself, and that convulsions and congestions should sometimes occur. The cerebral organ, at the time of birth, is said to be very crude and imperfect, and as it usually attains to completeness in its parts in about a year, the metamorphosis must be very rapid, and the vital energy exerted very great. Add to these conditions any disturbances of a sympathetic or local nature, among which may be classed those produced by teething, and the transition from health to disease, in this sensitive organ is easy. These above cited morbid conditions, have been mentioned as being among the most fatal of all the diseases accompanying dentition. The nervous system is usually involved in these disturbances when dentition does not proceed normally; but as it is not my present purpose to attempt a description of all the phenomena of dentition, my comments must be brief. Suffice it to say, that the latter manifestations are characterized in their mildest forms, by spasmodic motions of the face, eyes, arms, etc. If extending in their effects to the brain, perhaps, in combination with febrile or other symptoms, they may induce restlessness, sleeplessness and fitful slumbers, with their attendant *little horrors*, such as frightful dreams, etc., or if they become violent, may lead to very serious consequences. The most common of the local diseases, are those of an inflammatory nature, the effects being a fullness and tenderness of the gums, accompanied by heat and fever, the consequence of an increased flow of blood to the parts involved. These symptoms may vary in intensity, or disappear altogether to return again, either in a greater or less degree, when the interval between the first and second stages of the process shall have elapsed—the former, being considered the period when the absorption of the margins of the alveolus produced by the pressure of the crown takes place, and the latter, when the tooth having erupted therefrom, makes its way through the gum.

But to quote the words of Dr. Ellis, "Teething in the sense we are now considering, does not encompass all those beautiful and intricate processes concerned in the evolution and development of the germ, but is conventionally restricted to the period when the teeth and surrounding tissues are involved in those mutations designed to liberate the organs from their coverings, etc."

It is only in cases of difficult or retarded dentition, or when the diseases to which the infant may be predisposed are excited by the process, or the local structures are involved in diseased action, that any interference is called for, and then the treatment may be constitutional or surgical, one or both, according to the requirements of the case.

Most writers whom I have consulted, are agreed, that under certain conditions, incising the gums is productive of benefit. What, in brief, are those conditions?

First. When local depletion is required in order to relieve the pain produced by inflammation, as seen in the tumid, tense and injected gums.

Second. When the gum is prominent and distended over the advancing tooth, as indicated by its membranous appearance, so as to cause unfavorable symptoms by the local irritation.

Third. *When the tooth seems retarded in its approach to the surface, while the condition of the diseased gum remains unchanged and prolonged, general or constitutional disturbance is the effect; no abatement being had for days or weeks.

Fourth. When, during the process of eruption of a tooth, catarrh, or diarrhœa, or fever is excited, and ceases immediately on the piercing of the gum by the imprisoned organ.

Fifth. If, when the process of dentition is progressing rapidly, convulsions ensue without any apparent cause. When our object is to free the tooth from its investment, the incision must be of sufficient depth to sever the gum and capsule over the crown. If an anterior tooth, it must be made transversely—near the cutting edge, but over the labial surface, until the instrument reaches the enamel, which may be distinguished by a grating sound, and sometimes by a crackling report, on the sudden rupture of the investing membrane, as when the tension is severe. If a molar, the incision may be crucial, and directly over and through to the enamel and grinding surface of the crown, or it may be made from

* For the third, fourth and fifth condition, as above stated in substance, see West on the Diseases of Infancy and Childhood.

the anterior lingual to the posterior buccal cusp, and from the anterior buccal to the posterior lingual cusp, or vice versa. When our object is to reduce the inflammation, the scarification should be superficial, though a repetition is sometimes necessary. Great relief is often afforded by this local treatment—and Condie, Harris, Tomes, Dewees, Bond, West and others, unite in a recommendation of it under proper conditions, and when occasion calls, though doubtless, it is sometimes carried to excess.

The objection that lancing the gums retards the process of absorption, owing to the induration of the incised parts, has long ago been found to be without force. Also, that injury to the enamel of the tooth is likely to be caused by contact with the blade of the instrument, is answered by the fact, that the enamel is already hardened sufficiently at the time proper for the operation, to resist all injury from such a source. That it is a barbarous practice is without weight, for the pain produced is slight, while the relief afforded is immediate. Time will not admit of a consideration of the constitutional treatment necessary in cases involving the sympathetic diseases of dentition, neither is it called for at the present time; but, if this period is thus shown to be not unfrequently attended with so much pain and danger, while it has such an influence upon the future of the organs with which the dentist has to deal, ought it not to be to *him* an era of peculiar interest? As the perfectly organized body is dependent in a great measure, if not wholly, upon the proper performance of every physiological function, all means possible should be adopted to prevent an occurrence of those causes which militate against the normal processes of development of the teeth and osseous system, which being so closely allied in structure, draw their producing material from the same source.

Among the most important of these means, may be mentioned proper diet, exercise, cleanliness, pure air and an equable temperature. Imperfect formation of the teeth and maxilla, are very likely to coexist, as well as *protracted* development of the teeth and osseous system, and if the *former* appear in regular order, both as to time and place, they afford the best evidence of a normal development of the *latter*. "Protracted teething, retardation of the closure of the fontanels, and retardation of walking, usually go hand in hand," says Jacobi, and according to Eichmann and others "the large fontanel has finished its entire and permanent ossification," about the time of the completion of first dentition.

There are certain average periods, in which the eruption of the teeth takes place, commencing with the temporary about the fifth month of infancy, and terminating from the twenty-fourth to the thirty-sixth, and though there is considerable variation from these periods, healthy children are supposed to observe a certain degree of regularity in the process.

Eichmann. *As the result of four hundred observations on dentition, gives the following as the average time for the appearance of the deciduous teeth: The inferior incisors, from the seventh to the eighth month of infancy ; the superior incisors, from the ninth to the tenth; the anterior molars, from the twelfth to the thirteenth; the canines, from the sixteenth to the eighteenth, and the posterior molars, from the twenty-second to the twenty-fourth month. He reports a few in which the first tooth did not appear until the twenty-second month. I know of a German child who cut no teeth until twenty-two months of age, and in that case dentition was completed within twenty four months from birth.

Cases of long delayed dentition are not wanting, though fortunately rare, such as that reported by Merei, †of a child who, at the age of six years was toothless, the large fontanel closing at four, and one observed by Churchill, ‡of a child whose first teeth did not appear until the seventh year.

---

* Jacobi: On the Irregular Development of the Infantile Cranium.

† A. Schoeff Merei: On the disorders of Infantile Development, etc. London, 1855.

‡ Fleetwood Churchill, M. D.: Diseases of Infants and Children.

How are we to account for the retardation of the appearance of the temporary teeth, in cases like the foregoing? Is the blood deficient in bone producing material, and is the eruption of the teeth dependent upon a change in its constituents, which shall effect a deposition of lime salts in sufficient quantity to build up these structures? This would seem to be the case if the absorption of the alveolar margins, and gums confining the teeth, is dependent, as is generally thought, upon the pressure exerted thereon by these organs in the process of their general growth and development, as in the enlargement of the crowns and lengthening of the roots. In the case cited by Merei, the fact that the bones of the cranium were late in their development, as well as the teeth in their appearance, would also seem to favor the conclusion of a deficiency in the system, of ossific matter, as a cause for the late appearance of the latter. In regard to the absence of a single temporary tooth, usually present, Tomes says: "We are as little able to account for it, as to determine why twenty, rather than a smaller or greater number constitutes the normal series." Concerning the explanation of the latter phenomena, the *absence* of a single member from the usual number, we must, of course, look for a different explanation than the one just given for the *retardation* of the eruption of *all* the teeth to a late period, as a deficiency in any of the elements of which tooth structure is composed, would not be likely to be confined in its effects, to the space left vacant by the absence of the missing organ. In those very rare instances of persons who from infancy to life's close were edentulous, it would be gratifying to know whether there existed a normal or abnormal, a perfect or imperfect development of the osseous system generally.

But this paper is too long—longer than was anticipated at the commencement, though I have for the sake of brevity, viewed my subject from a few stand-points only.

That anything new should have been presented, by one who makes no pretention to anything more than very modest attainments in this branch of study, could not be expected. But as the organized body—the temple of God—made in His image, like an edifice of fair and beautiful proportions, may be seen and faintly comprehended as a whole, and yet not in all its aspects, because every vantage ground is not, or can not be appropriated, or some projecting angle, or cornice or column, hides from the eye many beauties of design or finish, which combine with these to form the perfect exterior, and while your soul must enter, would you behold the more wonderful adornments and luminous inscriptions within, so must it be with every mystery of life, as you gaze from every rising slope, and enter so far as may be into the spirit and interest of the subject, guided by the lights which have been placed about you, by the labors and the struggles of those who have gone before.

The mountain top must be reached before "all the kingdoms in the world" of vision, shall be spread out before us, with their glittering splendors. But how many have gained the summit? None! Only the infinite mind can fathom the mysteries of being, and the all-seeing eye penetrate the darkness to an unclouded day.

But, glimpses even may serve as encouragement.

Truth never wears out, and knowledge, like the diamond, increases in brilliancy by friction with its own disintegrated particles. Let us be "ever ready to come to a *knowledge* of the truth." Let no criticisms be withheld, for by such—kindly given and received, is it, that a healthy stimulus is afforded to the sluggish intellect, and aspirations excited that may add tone and vigor thereto, though those aspirations be not fully realized.

Much will be accomplished, should they lead to a more thorough investigation among members of the profession, of a subject which presents an ample and inviting field of labor.

